# Development of genomic phenotype and immunophenotype of acute respiratory distress syndrome using autophagy and metabolism-related genes

**DOI:** 10.3389/fimmu.2023.1209959

**Published:** 2023-10-23

**Authors:** Feiping Xia, Hui Chen, Yigao Liu, Lili Huang, Shanshan Meng, Jingyuan Xu, Jianfeng Xie, Guozheng Wang, Fengmei Guo

**Affiliations:** ^1^ Jiangsu Provincial Key Laboratory of Critical Care Medicine, Department of Critical Care Medicine, Zhongda Hospital, School of Medicine, Southeast University, Nanjing, China; ^2^ Department of Critical Care Medicine, The First Affiliated Hospital of Soochow University, Soochow University, Suzhou, China; ^3^ Department of Clinical Infection Microbiology and Immunology, University of Liverpool, Liverpool, United Kingdom

**Keywords:** autophagy, metabolism, genomic phenotype, immunophenotype, ARDS

## Abstract

**Background:**

Distinguishing ARDS phenotypes is of great importance for its precise treatment. In the study, we attempted to ascertain its phenotypes based on metabolic and autophagy-related genes and infiltrated immune cells.

**Methods:**

Transcription datasets of ARDS patients were obtained from Gene expression omnibus (GEO), autophagy and metabolic-related genes were from the Human Autophagy Database and the GeneCards Database, respectively. Autophagy and metabolism-related differentially expressed genes (AMRDEGs) were further identified by machine learning and processed for constructing the nomogram and the risk prediction model. Functional enrichment analyses of differentially expressed genes were performed between high- and low-risk groups. According to the protein-protein interaction network, these hub genes closely linked to increased risk of ARDS were identified with CytoHubba. ssGSEA and CIBERSORT was applied to analyze the infiltration pattern of immune cells in ARDS. Afterwards, immunologically characterized and molecular phenotypes were constructed according to infiltrated immune cells and hub genes.

**Results:**

A total of 26 AMRDEGs were obtained, and CTSB and EEF2 were identified as crucial AMRDEGs. The predictive capability of the risk score, calculated based on the expression levels of CTSB and EEF2, was robust for ARDS in both the discovery cohort (AUC = 1) and the validation cohort (AUC = 0.826). The mean risk score was determined to be 2.231332, and based on this score, patients were classified into high-risk and low-risk groups. 371 differential genes in high- and low-risk groups were analyzed. ITGAM, TYROBP, ITGB2, SPI1, PLEK, FGR, MPO, S100A12, HCK, and MYC were identified as hub genes. A total of 12 infiltrated immune cells were differentially expressed and have correlations with hub genes. According to hub genes and implanted immune cells, ARDS patients were divided into two different molecular phenotypes (Group 1: n = 38; Group 2: n = 19) and two immune phenotypes (Cluster1: n = 22; Cluster2: n = 35), respectively.

**Conclusion:**

This study picked up hub genes of ARDS related to autophagy and metabolism and clustered ARDS patients into different molecular phenotypes and immunophenotypes, providing insights into the precision medicine of treating patients with ARDS.

## Introduction

Acute respiratory distress syndrome (ARDS) is a profound pulmonary inflammatory reaction resulting from multiple potential causes associated with extremely high morbidity and mortality (1–3). ARDS is a global health problem, accounting for 10.4% of the admissions to ICU, and its mortality rate was about 30% to 60% (1). There is no specific treatment proved to be effective in decreasing the mortality or morbidity of ARDS (4) because ARDS is a heterogeneous syndrome rather than an explicit pathological disease.

Heterogeneity in ARDS is increasingly recognized as a principal barrier to developing efficacious targeted therapies. Phenotyping schemas are considered effective approaches in identifying a homogenous population within heterogeneous patients based on a multitude of data. In recent years they have been used in patients with ARDS to thoroughly understand the patients’ conditions and identify potential treatments (5). Further, a recent study has demonstrated that the ability to detect heterogeneity in ARDS diminished when phenotyping schema relied on only clinical data (6). Nevertheless, plenty of excellent types of research (7–10) have leveraged multivariate clinical data to phenotype patients of ARDS. On the other hand, trigging of ARDS depends on immune cells, such as neutrophils, monocytes, and macrophages, which propagate uncontrolled inflammation and tissue injury by secreting cytokines (11). Subsequently, adaptive immune responses are involved in the immunopathology of ARDS (12). Hence, recognizing different biological phenotypes (molecular phenotype and immunophenotype) has important implications for the precision medicine of patients with ARDS.

Autophagy is an evolutionarily conserved, lysosomal degradation pathway that is associated with cellular recycling, homeostasis, and elimination of intracellular pathogens (13). Autophagy controls inflammation through regulatory interactions with innate and adaptive immune signaling pathways, eliminating endogenous inflammasome agonists and immune mediators (14, 15). Recently, researchers have indicated that autophagy is essential in regulating the outcome of ARDS (16, 17). The fundamental essence of autophagy lies in its capacity to adapt to metabolic demands (18). Meanwhile, the metabolism data generated from clinical and experimental studies (19, 20) demonstrate a disturbance in energy and oxidative stress metabolism, which is consistent with the pathology of ARDS. The pathological alterations observed in ARDS may arise from a combination of genetic predisposition and immune response, leading to subsequent modifications in the downstream metabolites implicated in the development of ARDS. Therefore, host-derived metabolites are essential to connect to the pathogenesis of ARDS. Above all, we speculate that the phenotype classification of ARDS based on the characteristics of autophagy and metabolism is of great significance for its precision treatment.

In this study, we aimed to untangle the heterogeneity of ARDS based on autophagy and metabolism by using molecular and immune data in an algorithm of computation to determine homogenous phenotypes, with the aim to provide evidence in support of the concept of precision medicine of treating ARDS.

## Materials and methods

### Downloading and processing of data

Data containing three transcription profiles (GSE89953 (21), GSE32707 (22), and GSE76293 (23)) of acute respiratory distress syndrome (ARDS) and their corresponding clinical data were downloaded from the NCBI GEO database (24) (https://www.ncbi.nlm.nih.gov/geo/) from version 2.62.2 of ‘GEOquery’ package (25) in R software. The GSE89953 dataset, GSE32707 dataset, and GSE76293 dataset were based on GPL6883, GPL10558, and GPL570 platforms, respectively. The GSE89953 dataset comprised 26 acute respiratory distress syndrome (ARDS) patients. A total of 144 patients were included in dataset GSE32707, including 31 ARDS patients, 34 healthy controls, and 79 other patients, in which ARDS patients and normal patients were selected for this analysis. A total of 12 ARDS patients and 12 healthy controls of the dataset GSE76293 were included for analysis in the present study. The datasets GSE89953 and GSE32707 were set as the discovery cohort and GSE76293 as the validation cohort (**Table 1**). The datasets GSE89953 and GSE32707 were subjected to batch effect correction using the ‘sva’ (26) package in R. The Combined Datasets encompassed a total of 57 ARDS patients and 34 healthy controls.

**Table 1:** Acute respiratory distress syndrome data set information list.

	GSE89953	GSE32707	GSE76293
Platform	GPL6883	GPL10558	GPL570
Species	Homo sapiens	Homo sapiens	Homo sapiens
Tissue	alveolar macrophage	whole blood	Neutrophils
Samples in ARDS group	26	31	12
Samples in Normal group	0	34	12
Gender			
Male	15		
Female	11		
Age (years)			
Mean	41.8		
Median	46.5		
Reference	PMID: 28708019	PMID: 22461369	PMID:27064380

GEO, Gene Expression Omnibus; ARDS, Acute Respiratory Distress Syndrome.

HADb (Human Autophagy Database, http://www.autophagy.lu/index.html) is a public human autophagy-specific database. Autophagy-related genes (ARGs) were downloaded by HADb, and 222 ARGs were obtained (**Supplementary Table S1**). Metabolic-Related Genes (MRGs) were downloaded from the GeneCards (https://www.genecards.org/) database (27), with “metabolic” as the search keyword, after retaining MRGs with “Protein Coding” and correlation scores greater than 1, 7036 MRGs were obtained (**Supplementary Table S2**). A total of 159 autophagy and metabolism-related genes (AMRGs) were obtained after merging and deduplication (**Supplementary Table S3**).

### Identification of autophagy and metabolism-related differentially expressed genes

According to the grouping of the integrated GEO dataset (Combined Datasets), the patients were divided into the ARDS group and the normal group. The differential analysis of genes in the ARDS and normal groups was performed using the ‘limma’ package in R (28). Set |logFC| > 0.5 and adj. p< 0.01 as threshold for differential genes. Genes with logFC > 0.5 and adj. p< 0.01 are up-regulated differentially expressed genes, and logFC< -0.5 and adj. p< 0.01 are down-regulated differentially expressed genes. In order to obtain the AMRDEGs associated with ARDS, all |logFC| > 0.5 and adj. p< 0.01 differentially expressed genes (DEGs) in Combined Datasets and AMRGs take the intersection. The results of the differential analyses were displayed as a volcano map by the ‘ggplot2’ package, a heat map by the ‘pheatmap’ package, and a chromosome map by the ‘RCircos’ package (29) in R.

In order to explore the correlation between AMRDEGs, the Spearman algorithm was used. The TOP 3 positively and negatively correlated AMRDEGs are displayed by correlation scatter plots with the ‘ggplot2’ package in R. The ‘PROC’ package in R was used to plot the receiver operating characteristic (ROC) curve (30) of AMDEGs in the Combined Datasets to distinguish whether the patient is ARDS.

### Screening crucial AMRDEGs

To further screen the crucial AMRDEGs and candidate signatures, three machine learning [least absolute shrinkage and selection operator (LASSO) (31) logistic regression, support vector machine (SVM) (32), and random forest (RF) (33) algorithms were adopted. Based on AMRDEGs in the Combined Datasets, the LASSO logistic regression model with parameters seed = 3, family = “binomial” was run by the ‘glmnet’ package (34) in R and run 1000 cycles to prevent overfitting combined. SVM, a supervised machine learning technique, was executed with parameters seed = 3, method = “svmLinner” by the ‘caret’ package. AMRDEGs were screened with parameters seed = 3, ntree = 150 of a random forest model by ‘randomForest’ package. Finally, the genes at the intersection of those screened by LASSO, SVM, and random forest were used to diagnose ARDS.

### Constructing the nomogram and the risk prediction model

In order to analyze the diagnostic efficacy of crucial AMRDEGs for ARDS, we performed logistic regression analysis on crucial AMRDEGs in the Combined Datasets and constructed a logistic regression model. Based on the logistic regression analysis results, the ‘rms’ package was used to construct a nomogram (35). The calibration curve was used to assess the nomogram’s performance, and decision curve analysis (DCA) (36) was performed to evaluate the accuracy and resolution of the logistic regression model by the ‘rmda’ package.

To evaluate the predictive power of crucial AMRDEGs for ARDS, LASSO regression analysis was used to establish the prediction model. The Combined Datasets were used as the discovery cohort and the GSE76293 as the validation cohort. Risk scores were calculated from the expression of crucial AMRDEGs and LASSO regression coefficients.


$$\text{risk}Score\;={\;}_{\sum }^{i}Coefficient\;\left(gene_{i}\right)*mRNA\;Expression\;\left(gene_{i}\right)$$

According to the calculated mean risk score, patients were divided into high-risk and low-risk groups. The ROC curve was drawn to analyze the accuracy of the risk score in predicting whether the patient was ARDS by the ‘pROC’ package. The ‘ggplot2’ package was used to draw a risk scatter diagram to show the distribution of high and low-risk groups and the occurrence of ARDS. The expression of autophagy and metabolic phenotype-related crucial AMRDEGs in high and low-risk groups was shown in a hot map by the ‘pheatmap’ package. Taking the expression profile data of GSE76293 as the verification set, the risk scores were calculated by the above formulas. Grouping was based on the mean risk score, then the ROC curve, scatter plot and heat map were drawn.

### Screening differentially expressed genes and functional enrichment analysis

To identify genes associated with autophagy and metabolic phenotypes, according to the grouping of the high- and low-risk groups in the Combined Datasets, the ‘limma’ package of R was used to perform differential analysis. Set adjPvalue<0.01 and |logFC|>1 as the threshold to screen the genes, among which the genes with logFC>1 and adjPvalue<0.01 are the differentially expressed genes (DEGs) with up-regulated expression, and the genes with logFC<-1 and adjPvalue<0.01 are the genes with down-regulated expression. The results of the different analyses are displayed by drawing volcano maps and heat maps with the ‘ggplot2’ and the ‘pheatmap’ package.

Gene Ontology (GO) analysis (37) is a standard method for large-scale functional enrichment analysis, including biological process (BP), molecular function (MF), and cellular component (CC). The Kyoto Encyclopedia of Genes and Genomes (KEGG) (38) is a widely used database that stores information about genomes, biological pathways, diseases, and drugs. The ‘clusterProfiler’ package (39) was used to perform GO annotation analysis and KEGG pathway enrichment analysis on the differentially expressed genes of the high- and low-risk groups in the Combined Datasets. The false discovery rate (FDR)< 0.05 was considered statistically significant, and the screening standard was adjPvalue< 0.05 and q value< 0.05. The P value correction method is the Benjamini-Hochberg (BH).

In order to study the differences in biological processes between high and low-risk groups, we downloaded the “h.all.v7.5.1.symbols” gene set from the MSigDB database (40) and performed Gene set enrichment analysis (GSEA) (41). Adjusted P values less than 0.05 were considered statistically significant.

To further investigate the differences in biological processes between the high- and low-risk groups, the “h.all.v7.5.1.symbols” Gene Set was downloaded from the MSigDB database (40) to perform Gene Set Variation Analysis (GSVA) on the Combined Datasets (42). Adjusted P values of less than 0.05 were considered statistically significant.

### Identification of hub genes and construction of the related regulatory network

The protein interaction network is significant for understanding the functional connections between proteins (43). The STRING database (43) (https://string-db.org/) searches for proteins and predicts protein interactions. The database contains 67.59 million proteins and 20052.39 million protein-protein interactions. We used the STRING database to construct a protein-protein interaction (PPI) network for the differentially expressed genes of the high-risk group vs. the low-risk group in the Combined Datasets, and the coefficient was set to 0.4. The PPI results were exported from the STRING database and visualized by Cytoscape (44), and the hub genes in the PPI network were analyzed by CytoHubba (45).

miRTarBase (https://mirtarbase.cuhk.edu.cn/~miRTarBase/miRTarBase_2022/php/index.php) database (46) is a collection of microRNA-target interactions (MTI) database supported by experimental evidence. To study the regulatory relationship between hub genes and miRNAs, information about miRNA-mRNA interactions was downloaded from the miRTarBase database. Based on the hub genes obtained from the PPI analysis, the miRTarBase database was used to predict possible regulated miRNAs and construct a miRNA-mRNA regulatory network. Cytoscape software was used to visualize the miRNA-mRNA regulatory network.

Transcription factor (TF) controls gene expression by interacting with target genes at the post-transcriptional stage. TRRUST (Transcriptional Regulatory Relationships Unraveled by Sentence-based Text mining) (https://www.grnpedia.org/trrust/) is a human-annotated transcriptional regulation network database (47). Transcription factors that bind to hub genes were searched in the TRRUST database. The hub-TF network was subsequently visualized by Cytoscape.

### Immune infiltration analysis and construction of immunologically characterized phenotypes

Immune-related genes were downloaded from literature PMID:28052254 (48). The gene set contains 782 genes and 28 cell types. The degree of infiltration of immune cells was analyzed using the single-sample GSEA (ssGSEA) algorithm on the Combined Datasets by the ‘GSVA’ package (42). CIBERSORT (49) deconvolutes the transcriptome expression matrix based on the principle of linear support vector regression to estimate the composition and abundance of immune cells in mixed cells. The Combined Datasets were computed to derive the immune cell infiltration matrix by CIBERSORT. Boxplots were drawn to show the differences in the infiltration levels of 22 immune cells between the ARDS group and the Normal group in the Combined Datasets by the ‘ggplot’ package. For the immune cells with significant differences (P<0.05), the lollipop plot was used to show the correlation between immune cells and hub genes.

Consensus Clustering (50) could divide patients into several phenotypes according to different omics data sets to discover new disease phenotypes or conduct a comparative analysis. Cluster analysis was used by the ‘ConsensusClusterPlus’ package (51). According to the results obtained by ssGSEA, ARDS patients were divided into different groups according to the infiltration level of immune cells to construct immunologically characterized phenotypes. In this process, clusters were set between 2 and 8, and 80% of the total samples were extracted with 1000 repetitions, with clusterAlg = “km” and distance= “euclidean”.

### Construction of molecular phenotypes and correlation analysis with immune infiltrating cells

Molecular phenotypes of ARDS were identified using consensus clustering analysis based on ten hub genes using the ‘ConsensusClusterPlus’ package in R. According to the analysis results of ssGSEA. The ‘ggplot’ package was used to analyze the difference in the degree of immune cell infiltration among different phenotypes and the correlation between hub genes and immune cells in different molecular phenotypes.

### Statistical analysis

All data processing and analysis involved in this study were performed using R software (Version 4.1.1, https://www.r-project.org/). For comparisons of continuous variables between the two groups, the statistical significance of normally distributed variables was estimated utilizing an independent Student’s t-test, and differences between non-normally distributed variables were analyzed through the Mann-Whitney U test (i.e., the Wilcoxon rank-sum test). LASSO analysis was based on the glmnet package of R (34). All statistical *P* values are two-sided, and a *P* value of less than 0.05 was considered statistically significant.

## Results

### Identification of AMRDEGs between ARDS and control

**Figure 1:**
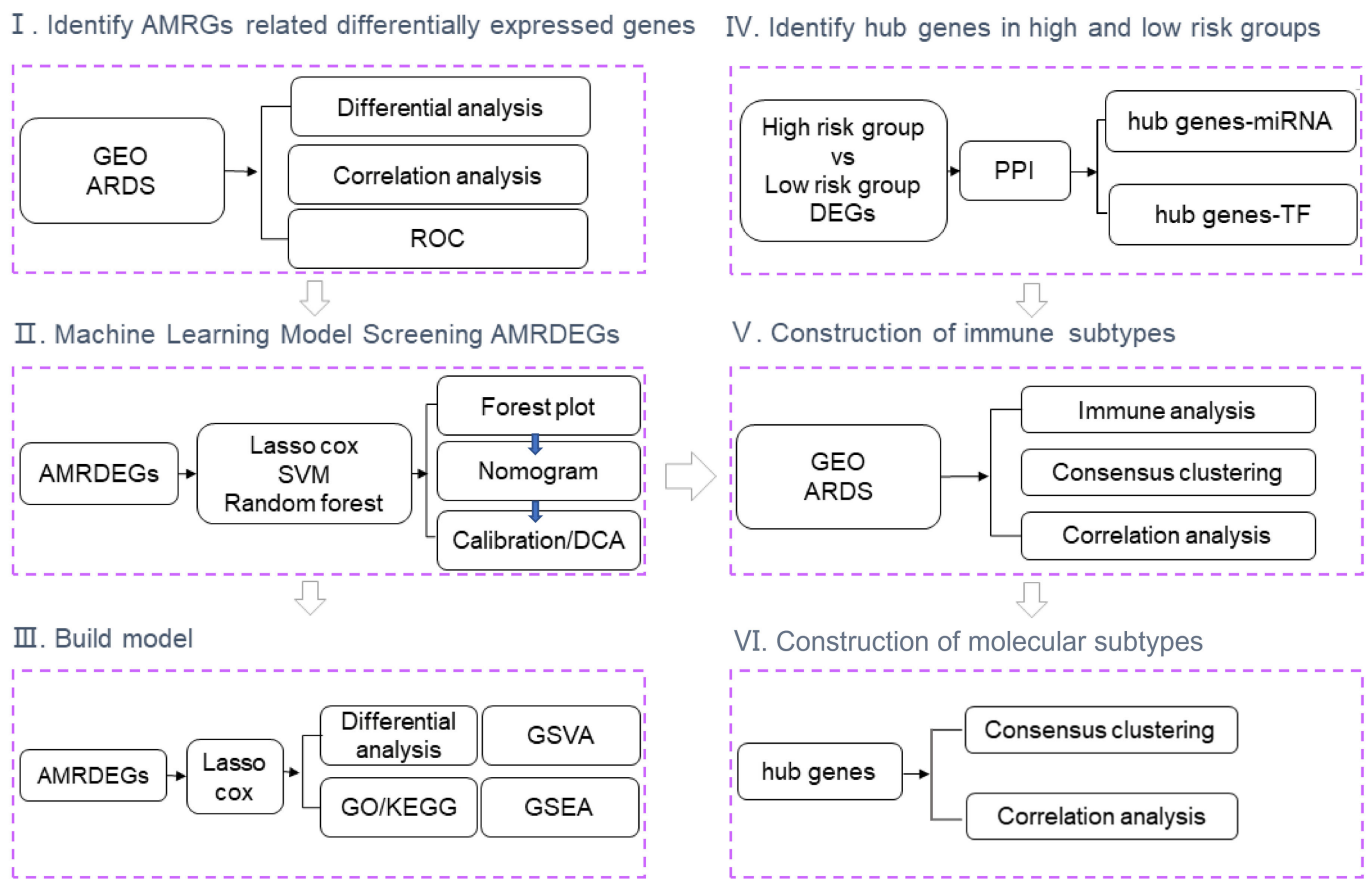
Flow chart of the study. GEO, Gene Expression Omnibus; ARDS, Acute Respiratory Distress Syndrome; ROC, Receiver Operating Characteristic; AMRDEGs, Autophagy And Metabolic-Related Differentially Expressed Genes; LASSO, Least absolute shrinkage and selection operator; SVM, Support Vector Machine; DCA, Decision curve analysis; DEGs, Differentially Expressed Genes; PPI, Protein-Protein Interaction Networks; TF, Transcription Factor; GO, Gene Ontology; KEGG, Kyoto Encyclopedia of Genes and Genomes; GSVA, Gene Set Variation Analysis; GSEA, Gene set enrichment analysis.

**Figure 2:**
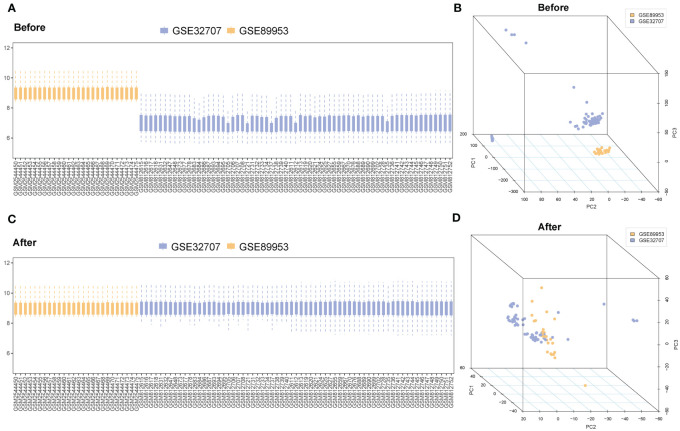
Merging and correction of databases. **(A)** The boxplot of the ARDS datasets before removing the batch effect. **(B)** Principal component analysis of the ARDS datasets before removing the batch effect. **(C)** The boxplot of the ARDS datasets after removing the batch effect. **(D)** Principal component analysis of the ARDS datasets after removing the batch effect. The blue represents the sample in the GSE32707; the orange represents the sample in the GSE89953. ARDS, Acute Respiratory Distress Syndrome.

A flow chart of the study is shown in **Figure 1**. GSE89953 and GSE32707, two sets of ARDS datasets, were combined to obtain the integrated GEO dataset after removing the batch effect. The batch effect of samples within the integrated GEO dataset was effectively alleviated following the batch removal procedure (**Figures 2A, C**). The efficacy of batch effect removal was further confirmed through Principal Component Analysis (PCA), which demonstrated the substantial elimination of batch effects among samples from diverse sources within the integrated GEO dataset (**Figures 2B, D**).

**Figure 3:**
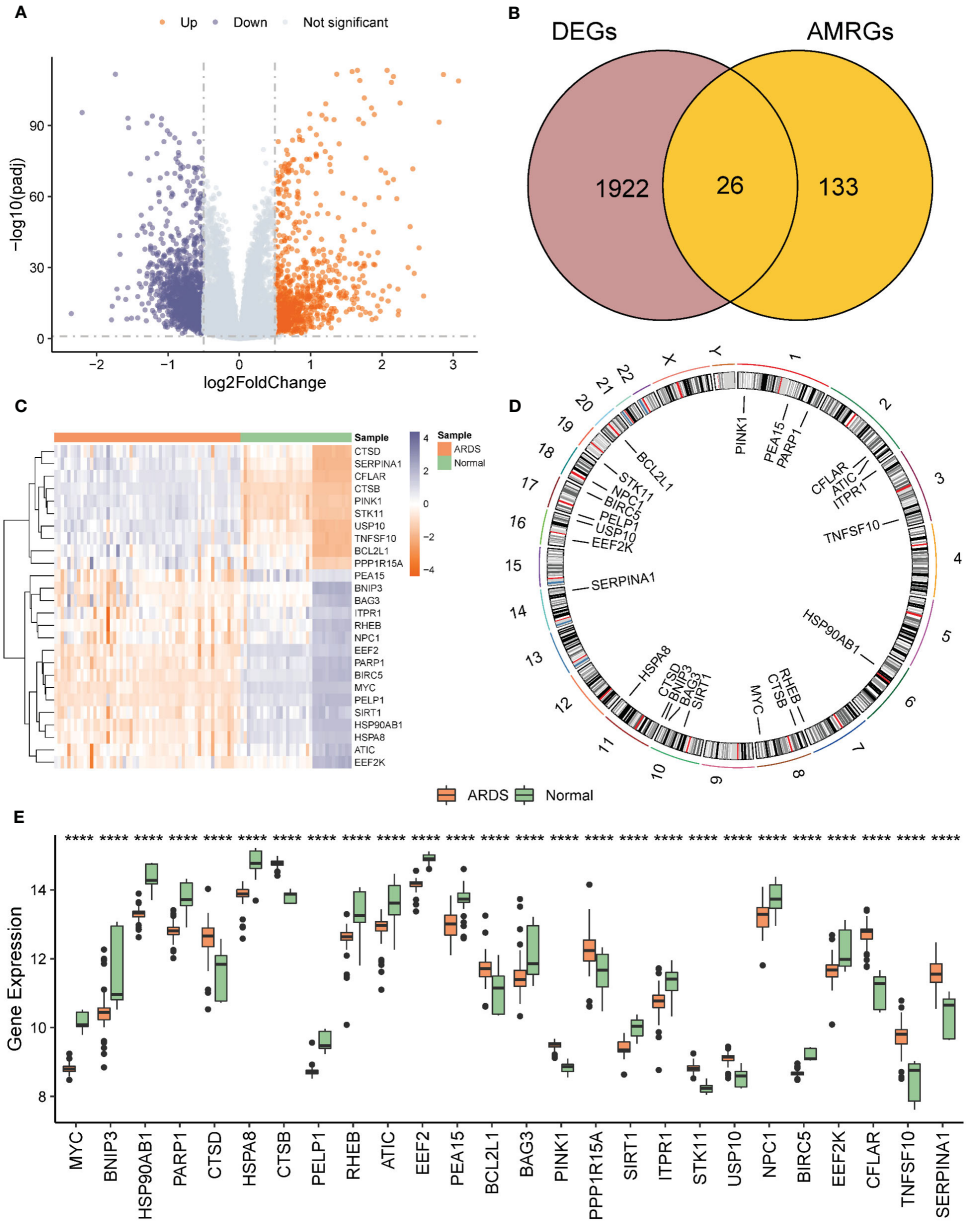
Analysis of differentially expressed genes related to ARDS. **(A)** Volcano of differential expression genes in ARDS group vs. normal group. A Differential expression volcano map of ARDS group vs. normal group. **(B)** Venn diagram of intersection of differentially expressed genes and AMRGS in ARDS vs. normal group. **(C)** Heat map of AMRDEGs expression in ARDS group vs. normal group. **(D)** Chromosomal map of AMRDEGs; **(E)** Boxplot of differential expression of AMRDEGs in ARDS group vs. normal group. **** represents p<0.0001. ARDS, Acute Respiratory Distress Syndrome; AMRGs, Autophagy And Metabolic-Related Genes; AMRDEGs, Autophagy And Metabolic-Related Differentially Expressed Genes.

**Figure 4:**
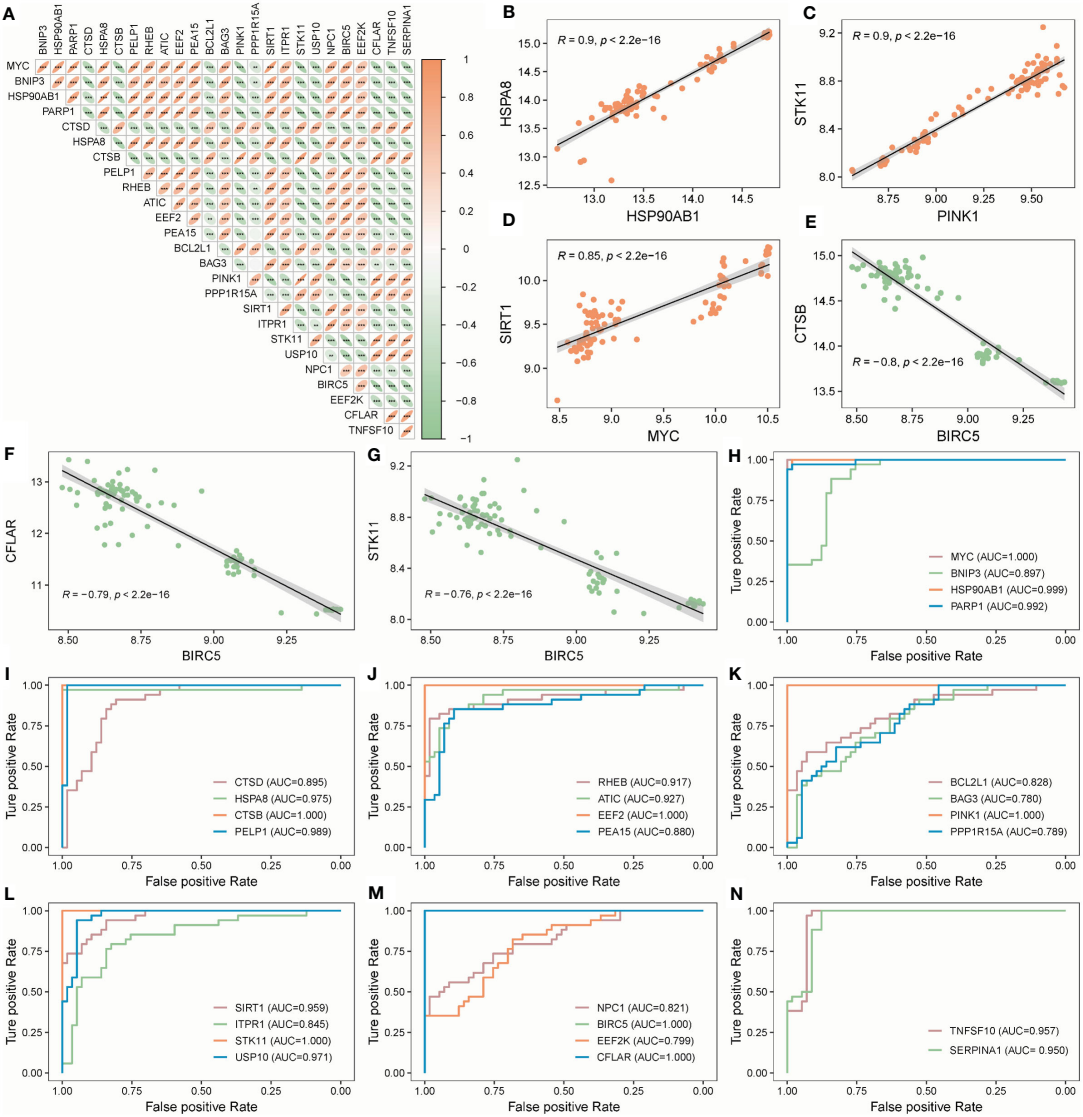
Correlation and ROC analysis of AMRDEGs in ARDS. **(A)** Correlation heatmap of 26 AMRDEGs in the Combined Datasets. **(B–D)** Scatter plot of correlation between TOP3 positively correlated genes. **(E–G)** Scatter plot of correlation between TOP3 negatively correlated genes. **(H–N)** ROC curves of 26 AMRDEGs in the Combined Datasets. ** represents p<0.01; *** represents p<0.001. AMRDEGs, Autophagy and Metabolic-Related Differentially Expressed Genes; ROC, Receiver Operating Characteristic.

26 AMRDEGs were obtained by the intersection of AMRGs and differentially expressed genes (**Figures 3A–C**). The location of AMRDEGs on human chromosomes was shown on the chromosomal map (**Figure 3D**). All AMRDEGs were significantly different between the two groups (P< 0.001) (**Figure 3E**). Further analysis for AMRDEGs in ARDS showed multiple correlations between the genes (**Figure 4A**). The degree of correlation was indicated by the r value. The synergistic effect was observed as the strongest between HSP90AB1 and HSPA8 (R = 0.901, P< 2.2e-16, **Figure 4B**), followed by STK11 and PINK1 (R = 0.897, P< 2.2e-16, **Figure 4C**), SIRT1 and MYC (R = 0.854, P< 2.2e-16, **Figure 4D**). In contrast, the competitive effect was found as the strongest between CTSB and BIRC5 (R = -0.802, P< 2.2e-16, **Figure 4E**), followed by CFLAR and BIRC5 (R = -0.786, P< 2.2e-16, **Figure 4F**), STK11 and BIRC5 (R = -0.762, P< 2.2e-16, **Figure 4G**). The ROC curve analysis revealed that 17 of the 26 AMRDEGs (**Figures 4H–N**) had high predictive accuracy, with a ROC curve above 0.9.

### Selection of crucial AMRDEGs via LASSO, SVM, and RF

**Figure 5:**
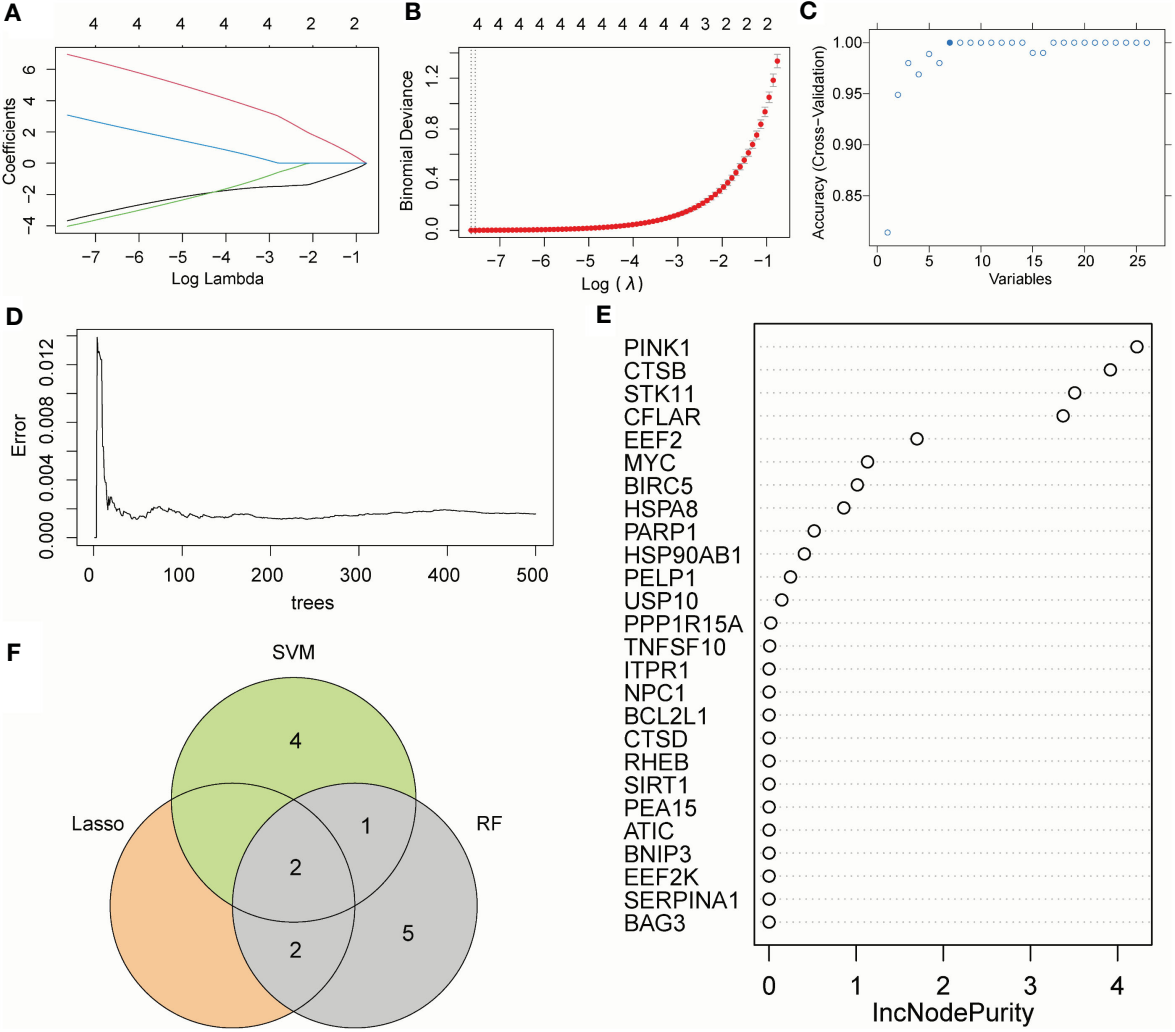
Machine learning algorithm for crucial AMRDEGs. **(A)** LASSO plot showed the variations in the size of coefficients for parameters shrank as the value of the k penalty increased. **(B)** Penalty plot of the LASSO model with error bars denoting standard errors. **(C)** Variable screening plot of SVM model. **(D)** The error rate confidence intervals for the random forest model. **(E)** The importance of genes in random forest model. **(F)** The interaction of the LASSO, SVM, and RF algorithms. AMRDEGs, Autophagy And Metabolic-Related Differentially Expressed Genes; LASSO, Least absolute shrinkage and selection operator; SVM, Support Vector Machine; RM, random forest; IncNodePurity, Increase in Node Purity.

To further optimize the screening of the crucial AMRDEGs, we applied three machine learning algorithms (LASSO regression, SVM-RFE, and RF). A total of 4 genes were screened by LASSO regression (**Figures 5A, B**). The accuracy of the SVM method reached its peak when the number of variables was 7 (**Figure 5C**). RF was used to select the top 10 genes ranked by variable importance (**Figures 5D, E**). By overlapping the three algorithms, two diagnostic signatures (CTSB and EEF2) were identified as crucial AMRDEGs (**Figure 5F**).

### Diagnostic efficacy and predictive power of crucial AMRDEGs in predicting ARDS

**Figure 6:**
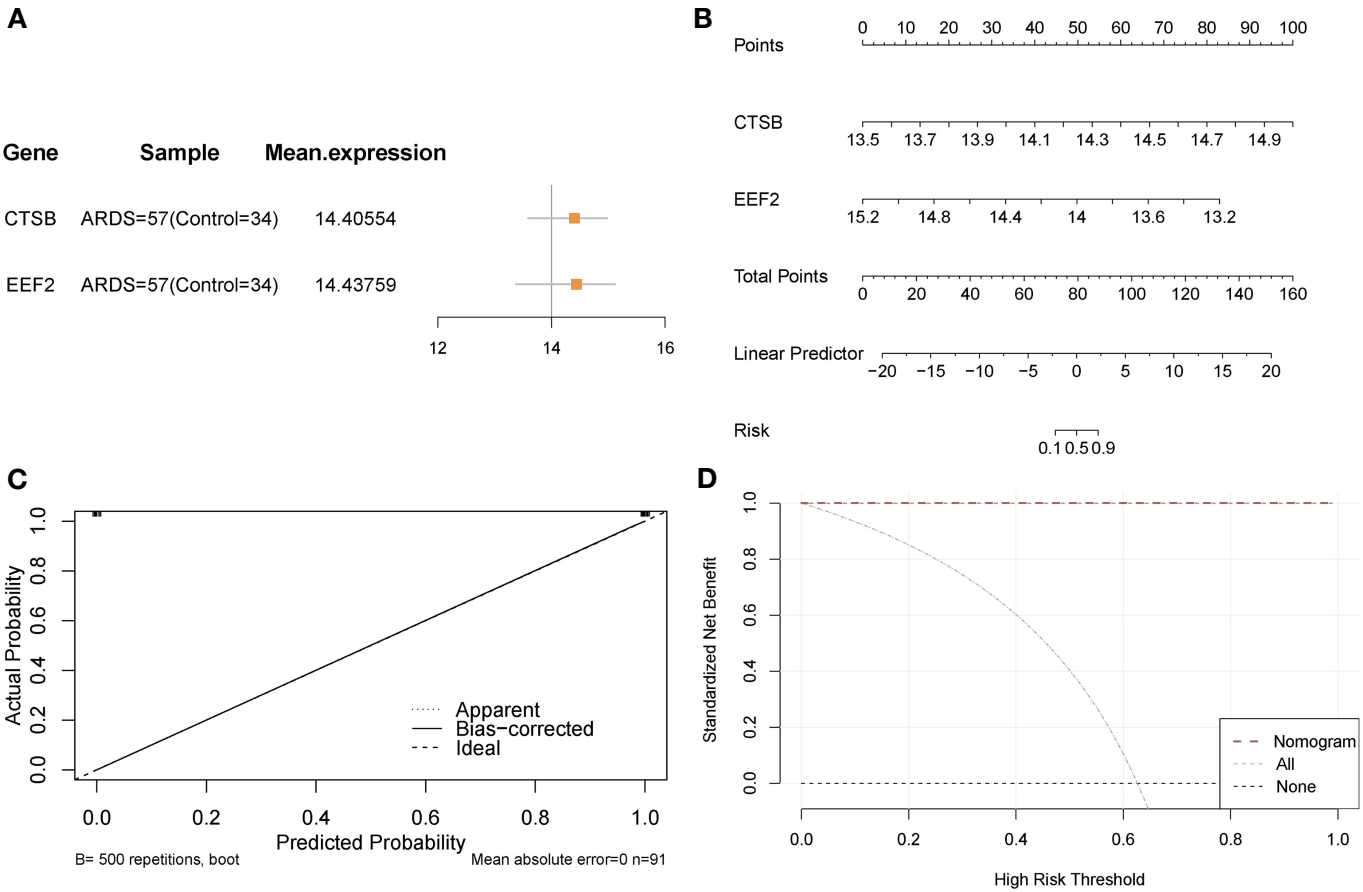
Validation of the diagnostic efficacy of crucial AMRDEGs. **(A)** forest plot of crucial AMRDEGs. **(B)** Nomogram showing the predicted risk for ARDS based on crucial AMRDEGs. **(C)** Calibration curve showing predicted performance of the nomogram. **(D)** DCA showing the clinical benefits of the nomogram. DCA, Decision Curve Analysis.

Expressions of CTSB and EEF2 were similar in the Combined Databases (**Figure 6A**). In addition, a predictive tool for ARDS development, a nomogram, was constructed by including these two signature genes related to autophagy and metabolism. In the nomogram, the value of each significant variable is associated with a score point, and the scores of all characteristic variables are summed to obtain the total score, which represents the risk of ARDS onset (**Figure 6B**). Calibration curves confirmed the accuracy of the nomogram in the diagnosis of ARDS (**Figure 6C**). DCA revealed that the application of the nomogram brought benefits to patients with ARDS (**Figure 6D**). Taken together, it can be concluded that the signature genes related to autophagy and metabolism show better diagnostic efficacy in predicting ARDS progression.

**Figure 7:**
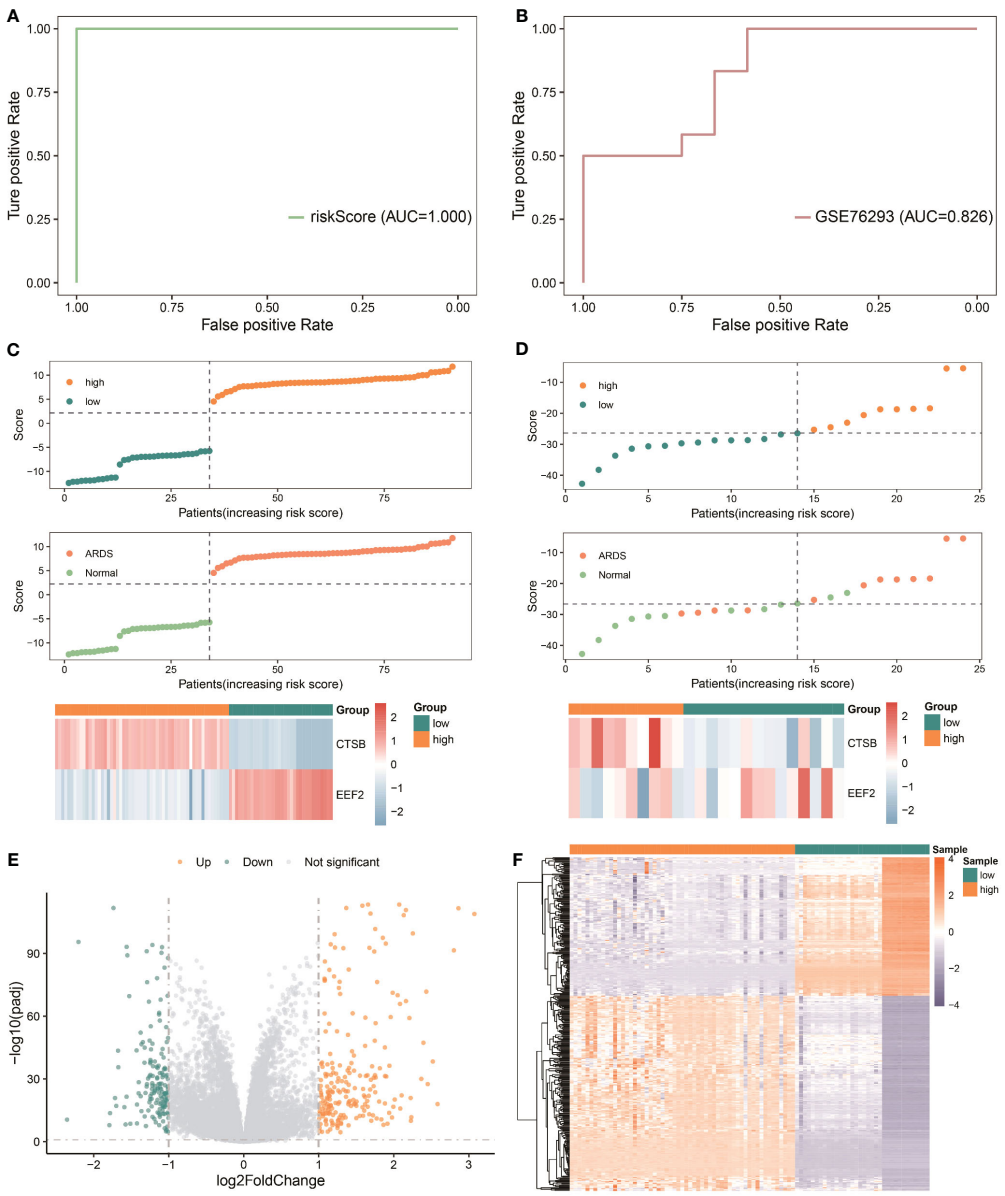
Predictive power of the risk model based on critical AMRDEGs and screening differential expression genes in the high-risk group of ARDS. **(A)** ROC curve of the risk score in the Combined Datasets. **(B)** ROC curve of the risk score in the validation set. **(C)** Distribution of risk scores, distribution of ARDS patients, and heat map of crucial AMRDEG expression in the integrated GEO dataset. **(D)** Distribution of risk scores, distribution of ARDS patients, and heat map of crucial AMRDEG expression in the validation set. **(E)** Volcano showed expression of differential expression genes between the high-risk and low-risk groups. **(F)** Heat map of differential expression genes expression in the high-risk and low-risk groups. ROC, Receiver Operating Characteristic; ARDS, Acute Respiratory Distress Syndrome.

In order to evaluate the predictive power of critical AMRDEGs in predicting ARDS, we integrated the expression of two diagnostic genes to construct a risk-scoring model. The analysis revealed that the risk score exhibited robust predictive capability for ARDS in both the discovery cohort (AUC = 1) (**Figure 7A**) and the validation cohort (AUC = 0.826) (**Figure 7B**). Then, the mean risk score (2.231332) was used to divide the patients in the discovery cohort into high- and low-risk groups. It was found that patients in the high-risk group were more inclined to be ARDS, CTSB was highly expressed in the high-risk group, and EEF2 was highly expressed in the low-risk group (**Figure 7C**). Finally, the patients in the validation cohort were divided into high- and low-risk groups using the mean value of the risk score (-25.53986), and the results showed the same finding (**Figure 7D**).

### Identification of differentially expressed genes of high and low-risk groups

The patients were divided into high- and low-risk groups to analyze the influence of the risk model on the occurrence and development of ARDS. A total of 371 differentially expressed genes were obtained, of which 214 genes were significantly up-regulated, and 154 genes were significantly down-regulated (**Figures 7E, F**).

**Figure 8:**
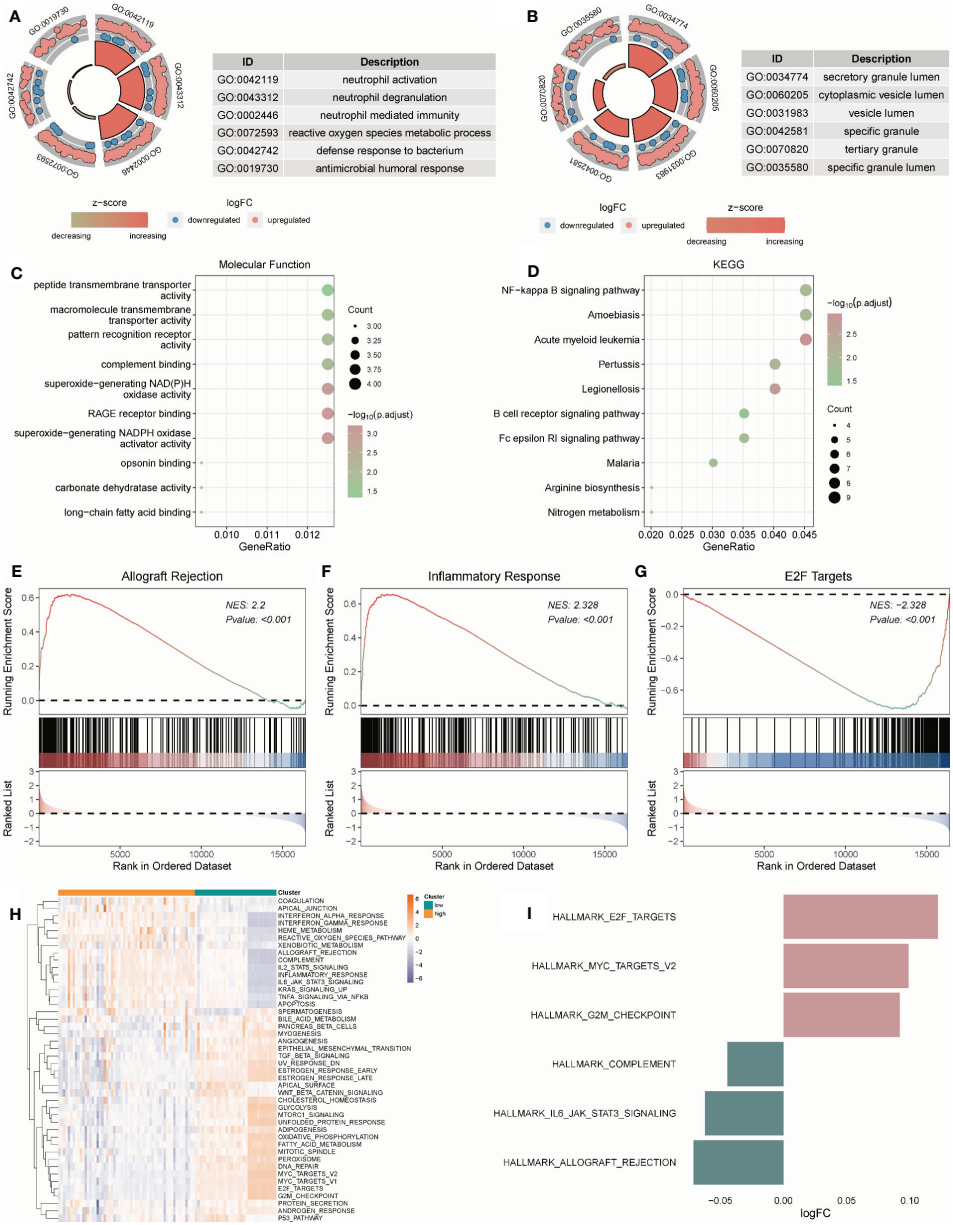
Functional enrichment analysis of genes in high and low-risk groups. **(A-C)** The functional enrichment in BP, CC, and MF analysis, respectively. **(D)** The KEGG analysis of differential expression genes. **(H, I)** GSVA-based analysis of biological function enrichment of hallmark gene set by heat map **(H)** and bar plot **(I)**. **(E–G)**: Allograft Rejection **(E)**, Inflammatory Response **(F)**, and E2F Targets **(G)** show three pathways related to high and low-risk groups. The abscissa is the gene, sorted according to the logFC value in the differential gene list, logFC greater than 0 indicates up-regulated genes are marked in red, logFC less than 0 indicates down-regulated genes are marked in blue, the upper ordinate is the enrichment score, and the lower ordinate is the logFC value. BP, Biological Process; CC, Cellular Component; MF, Molecular Function; KEGG, Kyoto Encyclopedia of Genes and Genomes.

Based on differentially expressed genes, GO, and KEGG analyses were performed. The results showed that the differentially expressed genes were mainly correlated with neutrophil activation (BP), secretory granule lumen (MF), peptide transmembrane transporter activity (CC), and NF-kappa signal pathway (KEGG) (**Figures 8A–D**; **Supplementary Tables S4-5**).

The gene expression in high- and low-risk patients were further processed for functional enrichment with GSEA and GSVA. GSEA showed that pathways such as allograft rejection (**Figure 8E**) and inflammatory response (**Figure 8F**) were significantly enriched in high-risk patients, and E2F targets (**Figure 8G**) were significantly enriched in low-risk patients (**Supplementary Table S6**). GSVA showed that the low-risk group was significantly enriched in E2F targets and MYC targets V2, while the high-risk group was significantly enriched in complement, IL6 JAK STAT3 Signaling and allograft rejection (**Figures 8H, I**).

### Selecting hub genes and construction of the related regulatory network

**Figure 9:**
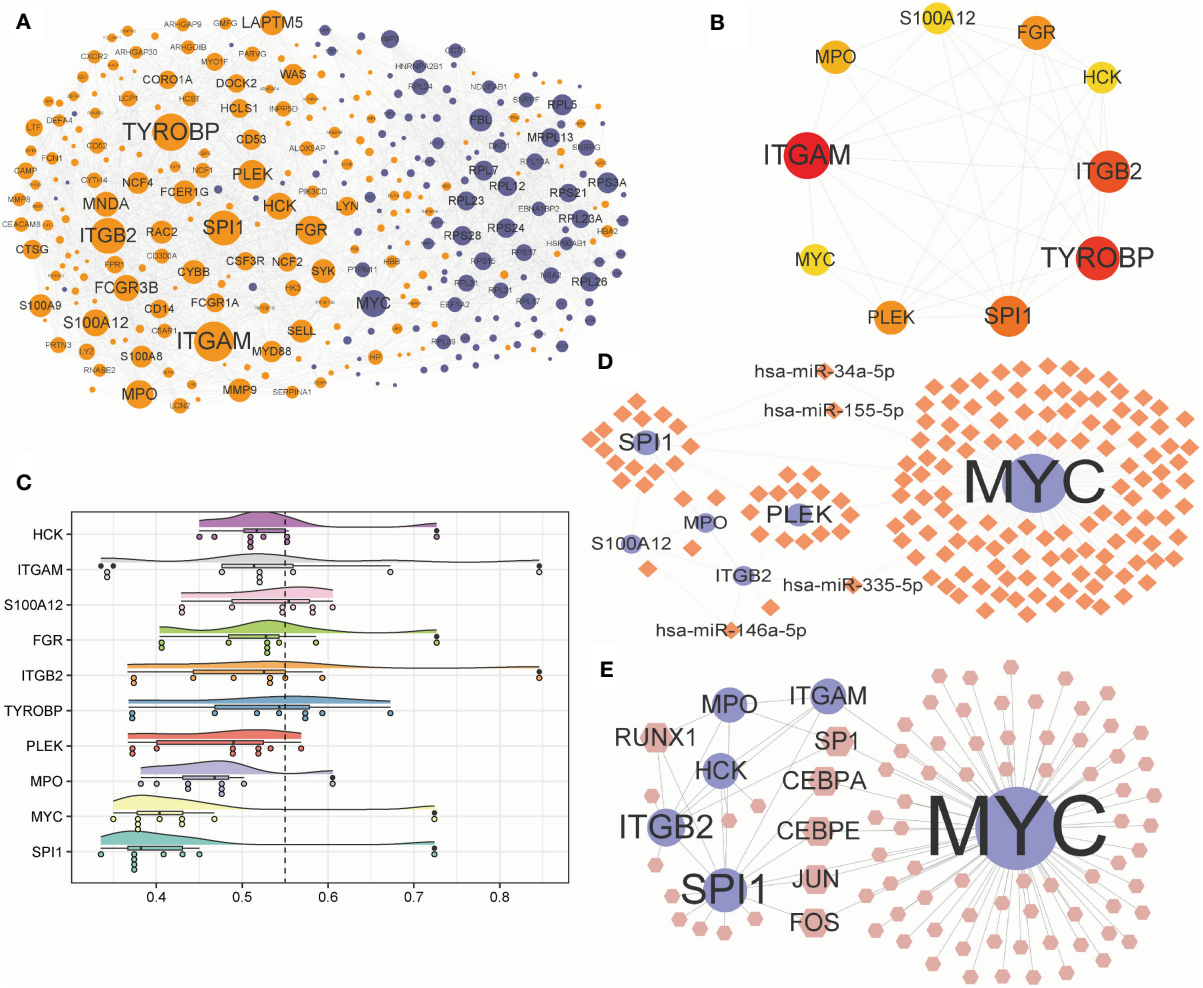
Construction of PPI network and interconnected regulatory network. **(A)** protein-protein interaction network constructed by differential expression genes. **(B)** Network of the top ten hub genes. **(C)** Cloud and rain map of hub gene importance. **(D, E)** miRNA-mRNA **(D)** and TF-mRNA **(E)** regulatory network constructed by hub genes. PPI, protein-protein interaction; TF, Transcription Factor.

PPI of 371 differential genes in high- and low-risk groups was analyzed using the STRING database and visualized as a network with the Cytoscape (**Figure 9A**). With CytoHubba, ten candidate hub genes were identified from the PPI network, including ITGAM, TYROBP, ITGB2, SPI1, PLEK, FGR, MPO, S100A12, HCK, and MYC (**Figure 9B**). Friends analysis found that the HCK gene played an essential role in the hub genes (**Figure 9C**). Further analysis showed that MYC, SPI1, and PLEK genes played significant regulatory roles in the miRNA-mRNA regulatory network (**Figure 9D**). For the TF-mRNA regulatory network, MYC, SPI1, and ITGB2 play essential roles in the regulation (**Figure 9E**).

### Analysis of hub genes and infiltrated immune cells

**Figure 10:**
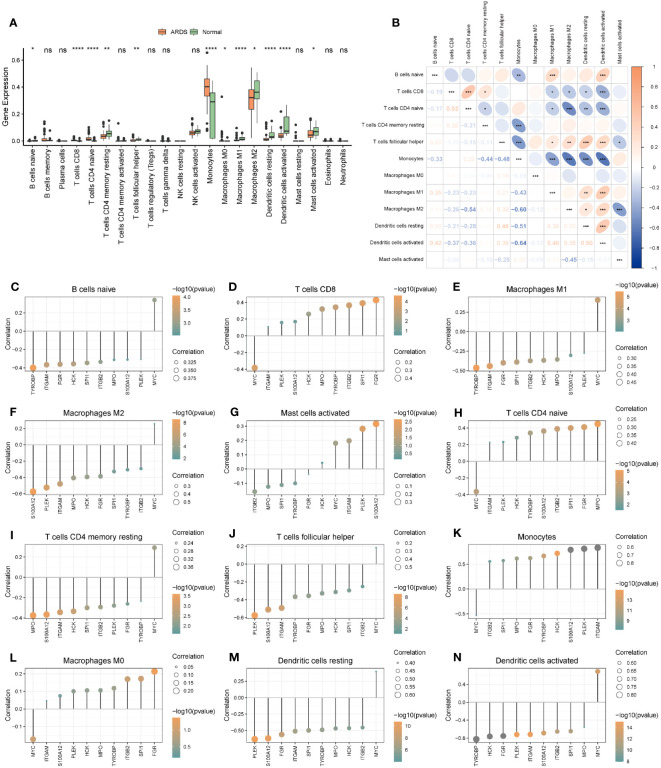
The immune cell infiltration and association with hub genes. **(A)** The immune cell infiltration in the ARDS and the Normal group. **(B)** Correlations of differently infiltrated Immune cells. **(C–N)** The association between hub genes and differently infiltrated Immune cells. * represents p<0.05; ** represents p<0.01; *** represents p<0.001; **** represents p<0.0001. ARDS, Acute Respiratory Distress Syndrome; ns, no significance.

For ARDS patients in Combined Datasets, levels of infiltration of immune cells were shown in **Supplementary Table S7**. Moreover, 12 of 22 infiltrated immune cells were differentially expressed between the ARDS group and the normal group in the Combined Datasets (**Supplementary Table S8**; **Figure 10A**). Most of the different immune cells were negatively correlated, among which the highest positive correlation was between T cells CD4 naive and T cells CD8 (r = 0.518), and the highest negative correlation was observed between dendritic cells activated and monocytes (r = -0.639) (**Figure 10B**). These correlations between different immune cells and hub genes are shown in **Figures 10C–N**.

### Construction of immunologically characterized phenotypes

**Figure 11:**
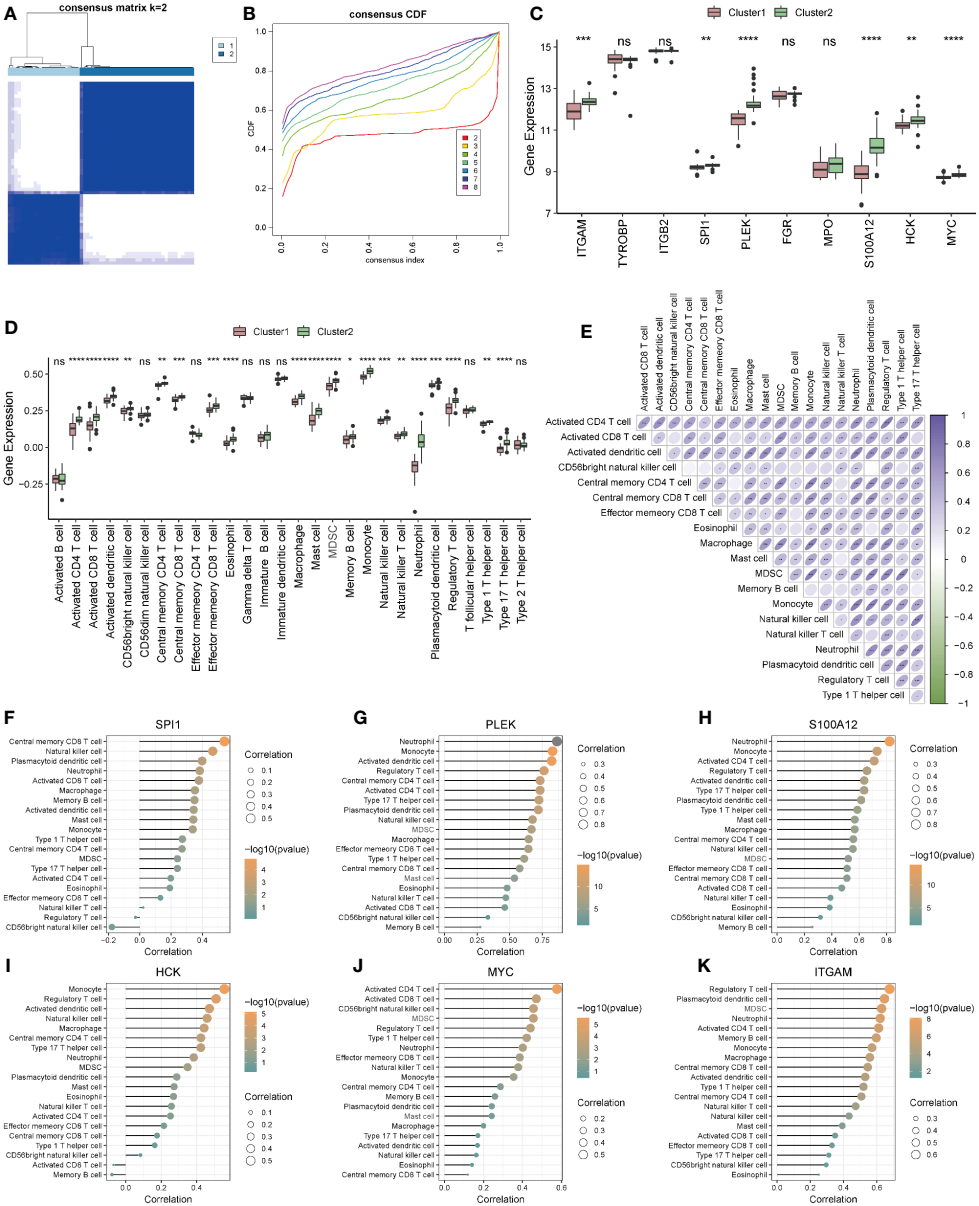
Construction and correlation analysis of immune signature phenotypes. **(A)** Consensus clustering matrix when k = 2. **(B)** Consensus CDF curves when k = 2 to 8. **(C)** Boxplot of hub genes in different immune phenotypes. **(D)** Boxplot of immune cells in different immune phenotypes. **(E)** Correlations of differential immune cells. **(F–K)**: Correlation between differential hub genes and differential immune cells. * represents p<0.05; ** represents p<0.01; *** represents p<0.001; **** represents p<0.0001. CDF, Cumulative Distribution Function; ns, no significance.

According to the analysis results of ssGSEA, ARDS patients in the Combined Datasets were divided into two different immune phenotypes (Cluster1: n = 22; Cluster2: n = 35) by unsupervised consensus clustering (**Figures 11A, B**; **Supplementary Table S9**). Next, we compared the differences in hub genes (**Figure 11C**) and immune cells (**Figure 11D**) in immune signature phenotypes, and analysis correlations of differential immune cells (**Figure 11E**). The results showed that there were six hub genes with significant expression differences (**Figure 11C**) and 20 kinds of immune cells with significant infiltration differences (**Figure 11D**) in immune signature phenotypes (**Figure 11C**). The heat map of correlations between differential immune cells shows that there is a positive correlation between immune cells, among which Plasmacytoid dendritic cell and Monocyte have the highest positive correlation (r=0.780, P=8.900e-13). Differential hub genes and immune cells have correlations (**Figures 11F–K**).

### Construction of molecular phenotypes and correlation analysis with immune infiltrating cells

**Figure 12:**
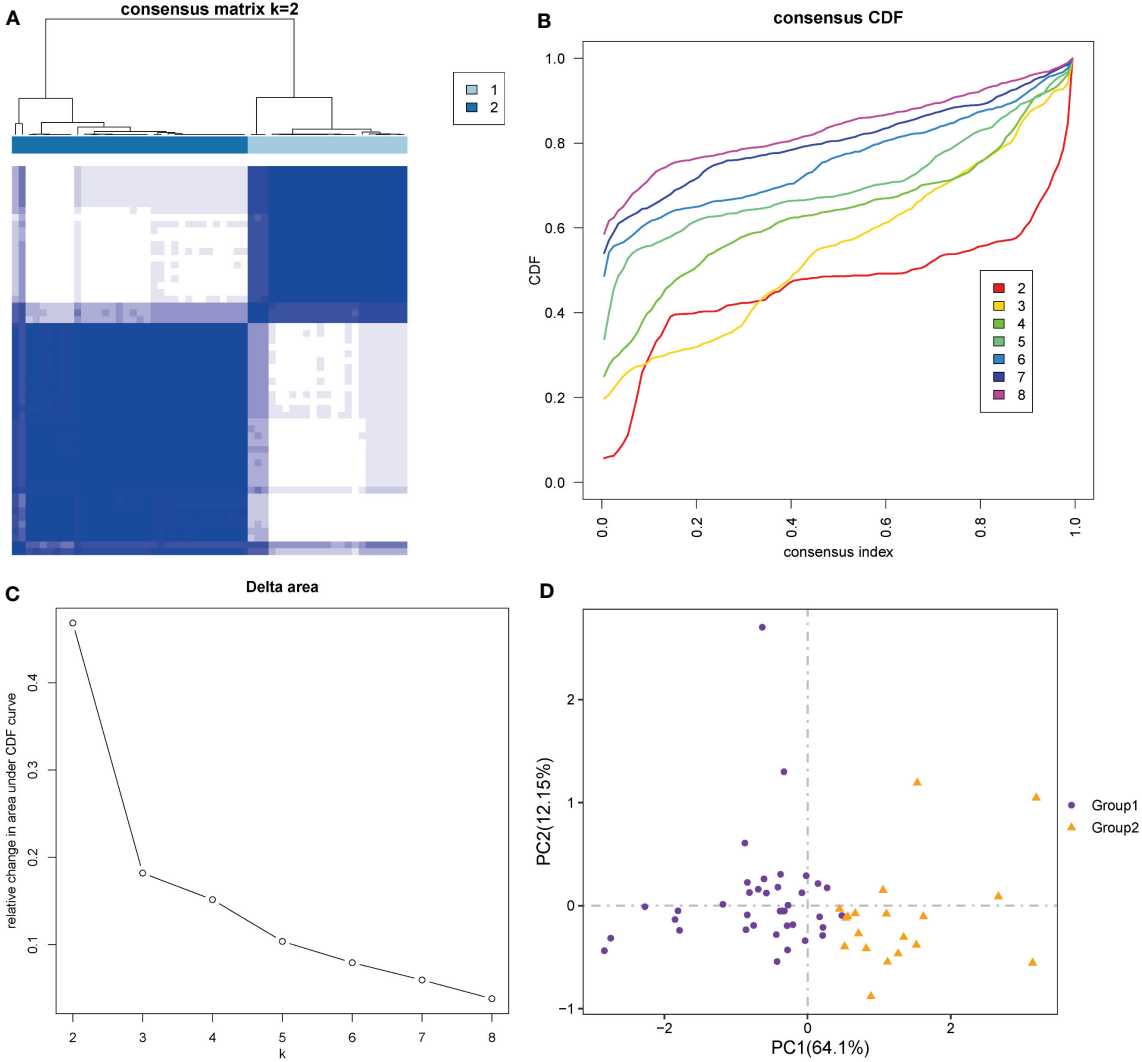
Construction of molecular phenotypes of ARDS. **(A)** Consensus clustering matrix when k = 2. **(B)** Consensus CDF curves when k = 2 to 8. **(C)** Relative alterations in CDF delta area curves. **(D)** Principal component analysis performed to distinguish Group 1 and Group 2. (CDF) Cumulative Distribution Function.

According to the ten hub genes, ARDS patients in the Combined Datasets were divided into two different molecular phenotypes (Group 1: n = 38; Group 2: n = 19) by unsupervised consensus clustering when the number of clusters is 2 (**Figures 12A–C**; **Supplementary Table S10**). The result of the principal component analysis shows that the two groups of ARDS patients could be well distinguished (**Figure 12D**).

**Figure 13:**
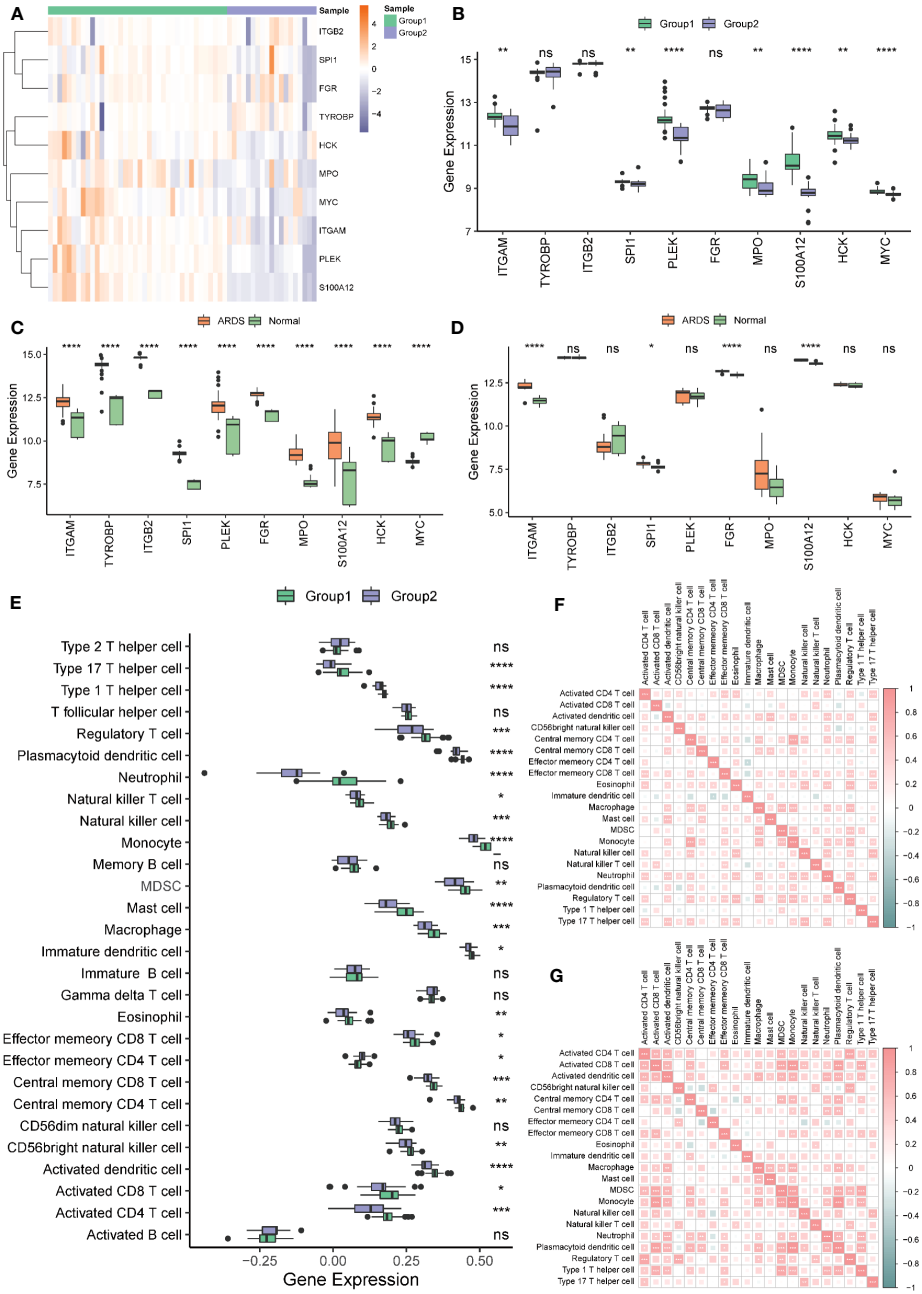
Hub genes and immune cell infiltration of molecular signature phenotypes. **(A)** Heatmap of hub genes expression in Group 1 and Group 2. **(B)** Boxplot of hub genes in Group 1 and Group 2. **(C, D)** Boxplot of hub genes between ARDS and Normal group in the Combined **(C)** and the GSE76293 dataset **(D)**. **(E)** Immune cell infiltration between Group 1 and Group 2. **(F, G)** The correlation of the immune cells in Group 1 **(F)** and Group 2 **(G)**. * represents p<0.05; ** represents p<0.01; *** represents p<0.001; **** represents p<0.0001. ARDS, Acute Respiratory Distress Syndrome; ns, no significance.

Most of the hub genes were highly expressed in Group 1 and under-expressed in Group 2 (**Figure 13A**). There were significant differences in most hub genes between the two subgroups, and the expression value in Group 1 was significantly higher than in Group 2 (**Figure 13B**). In both the Combined Datasets (**Figure 13C**) and GSE76293 datasets (**Figure 13D**), the expression values of most genes in the ARDS group were significantly higher than those in the normal group. Further analysis revealed that more than 70% of immune cells significantly differed between the two groups (**Figure 13E**). Both in Group1 (**Figure 13F**) and Group2 (**Figure 13G**), most of the differential immune cells were positively correlated.

**Figure 14:**
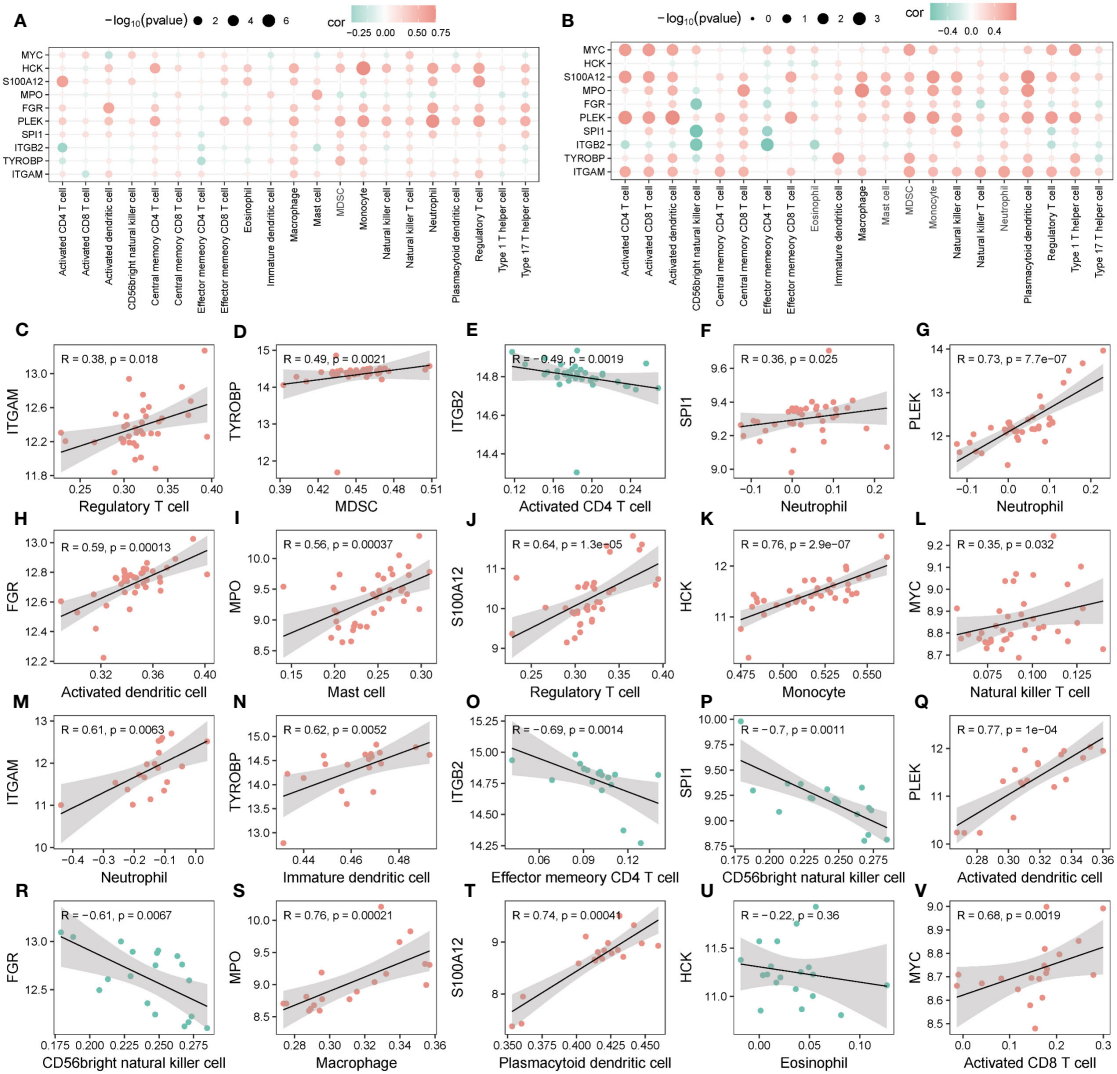
Correlation of hub genes and infiltrating immune cells. **(A, B)** Correlation between hub genes and immune cells in Group 1 **(A)** and Group 2 **(B)**. **(C-V)** Scatter plot of top 10 correlations between hub genes and immune cells in Group1 **(C-L)** in Group2 **(M-V)**.

Most hub genes positively correlated with immune cells in groups 1 (**Figure 14A**) and 2 (**Figure 14B**). The top ten correlations of genes and immune cells in Group 1 and Group 2 were shown in **Figures 14C–L** and **Figures 14M–V**, respectively.

## Discussion

The discrimination of ARDS phenotypes holds clinical significance in the context of devising tailored therapeutic interventions. However, the identification of phenotypes is currently mainly based on clinical indicators. In this study, we delved into the critical insights gained from analyzing autophagy and metabolism-related genes to identify and differentiate phenotypes of ARDS. We stratified patients into two groups, high-risk and low-risk for ARDS development, which represents a crucial step toward better patient management. Through in-depth bioinformatics analysis, we identified a set of ten candidate hub genes, namely ITGAM, TYROBP, ITGB2, SPI1, PLEK, FGR, MPO, S100A12, HCK, and MYC, with significantly altered expression. Additionally, we revealed two distinct phenotypes within the ARDS patient population, enhancing our understanding of the intrinsic heterogeneity of this syndrome.

In this study, we employed bioinformatics analysis to identify a panel of 10 hub genes, assessed the infiltration of immune cells using ssGSEA and CIBERSORT algorithms, and performed consensus clustering analysis to classify ARDS subtypes based on the identified hub genes and infiltrating immune cells. The crucial AMRDEGs associated with ARDS were identified by LASSO, SVM, and RF analysis, including CTSB and EEF 2. The application of three machine learning methods further ensured the reliability of screened genes. We then analyzed the diagnostic efficacy of crucial AMEDEGs for ARDS by constructing a nomogram. After that, a risk prediction model was established to evaluate the predictive power of ARDS according to the risk score calculated by the crucial AMERGs and was further verified in the validation cohort. Subsequently, we screened DEGs between the high- and the low-group and explored the function enrichment analysis based on GO, KEGG, GSEA, and GSVA. The hub genes associated with ARDS were selected by PPI analysis and explored the correlation with infiltrated immune cells. Finally, consensus clustering was applied to construct genomic and immune phenotypes, and the correlations between hub genes and immune cells were further analyzed in different phenotypes. Our findings underscore the pivotal involvement of autophagy, metabolism, and immunity in the underlying pathophysiological mechanisms of ARDS.

As a result of our study, we selected ten autophagy and metabolism-related hub genes. A total of 6 genes, ITGAM, SPI1, PLEK, S100A12, HCK, and MYC, were expressed differently between different phenotypes. ITGAM has been identified to encode integrin αM (CD11b). It’s a surface marker of monocytes and mediates various cell functions, including chemotaxis, adhesion, and transendothelial migration (52, 53). CD11b expressions were found enhanced in ARDS alveolar polymorphonuclear neutrophils (23), which is consistent with our study, that ITGAM was highly expressed in ARDS patients in both the Combined Datasets and the validation set. SPI1 encoding transcription factor PU.1 regulates the innate immune function of alveolar macrophages (54) and functions to initiate inflammatory cascade through activating alveolar macrophages (55, 56). The transcribed protein of PLEK has been identified in platelets and is involved in platelet biology (57, 58). There is currently a lack of data on the relationship between SPI1 and PLEK with ARDS. In our study SPI1 was highly expressed in ARDS patients, while PLEK was not consistently expressed in the two datasets. S100A12, known as a RAGE ligand, elicits a proinflammatory response in leukocytes and endothelial cells. High concentrations have been found in lung tissue and bronchoalveolar lavage fluid in acute lung injury (59), consistent with our study. HCK is a member of the Src family expressed in myelomonocytic cell lineages, ultimately affecting cellular proliferation, differentiation, and migration (60), and has played an essential role in ARDS (61–63). The MYC-encoded protein forms a heterodimer with the transcription factor and has different expressions in ARDS patients and controls (64). Our study showed that the expression of HCK and MYC was controversial in different datasets but was consistent in phenotype. These differences may be related to the heterogeneity of ARDS, which further illustrates the necessity of phenotypic classification of ARDS patients.

Differentially expressed genes in high- and low-risk groups have been identified. The high-risk group was significantly enriched in regulating immune inflammation, including neutrophil activation, inflammatory response, and complement. The related pathways in the high-risk group mainly focused on IL6-JAK-STAT3 signaling, NF-kappa signal pathway, and E2F targets were increased considerably in low-risk patients. The result revealed that immune inflammation may play a crucial role in the development of ARDS in high- and low-risk groups. This result is consistent with previous phenotypic classifications of ARDS (7, 9, 65). However, the previous phenotype classification based on inflammatory factors was limited to clinical markers, so it is necessary to classify phenotypes based on molecular mechanisms to achieve precise treatment of ARDS.

We subsequently evaluated the roles of hub genes in immune cell infiltration and constructed the immunophenotypes. After we had selected the ten autophagy and metabolism-related hub genes, it was found that they could be used to cluster patients with ARDS. Our research found that most of the hub genes and immune cells were highly expressed in cluster 2 and group 1. It means that cluster 2 and group 1 are mainly immune activations. It is consistent with the current classification of ARDS on the high and low-inflammation types (8, 9). Further research is needed to integrate the molecular, immune, and clinical features of ARDS to classify subtypes and guide the precise treatment of ARDS. Patients in the different clusters and groups had differently infiltrated immune cells, suggesting that autophagy and metabolism in patients with ARDS might regulate immune status, which might lead to a different prognosis.

Our study has limitations. First, all analyses were based on data obtained from public databases. GEO dataset is restricted in terms of species representation, sequencing platforms, molecular types, sample grouping, and sample quality. However, the availability of suitable datasets for our analysis was limited to the dataset used in this study. Although we have set the validated cohort, further validation would be best done in prospective studies. We have also emphasized our commitment to accumulating samples from our institution for sequencing, aiming to supplement the shortcomings of this study in our subsequent research. Additionally, we acknowledge the limitations of different sample types can lead to variations in gene expression profiles and potentially introduce noise if the biological relevance of sample types is not appropriately considered. Secondly, the results do not provide a comprehensive view of the role of autophagy in ARDS because the RNA sequence findings do not distinguish between phosphorylation and dephosphorylation of autophagy-related proteins. Proteomics analysis is required before translational application. Thirdly, it is imperative to conduct experimental investigations to delve deeper into the molecular mechanisms involving the interplay between autophagy, metabolism-related genes, and immune cells. Additionally, thorough experimental validation is warranted to ascertain the prognostic implications for patients exhibiting distinct ARDS phenotypes.

Our study identified ten candidate hub genes and classified ARDS patients into two distinct phenotypes. These findings hold direct clinical significance, paving the way for more precise diagnosis and personalized treatment strategies. Moreover, our research establishes a foundation for future experimental and clinical investigations, providing valuable directions for further exploration of ARDS and its therapeutic approaches. In future studies, the expression of the hub genes in ARDS patients can be explored in relation to clinical parameters such as disease severity and treatment response. Furthermore, we can delve deeper into the investigation of immune cells associated with the hub genes to better understand their roles in the pathogenesis of ARDS. Specifically, we can explore the distribution and functions of these immune cells in different phenotypes of ARDS. This may aid physicians in better guiding the treatment of ARDS patients.

## Conclusion

In conclusion, the present study picked up ten hub genes of ARDS related to autophagy and metabolism, namely ITGAM, TYROBP, ITGB2, SPI1, PLEK, FGR, MPO, S100A12, HCK, and MYC, which could cluster ARDS patients into different molecular phenotypes. Besides, we also explored the infiltrated immune cells of ARDS and clustered ARDS patients into different immunophenotypes. We further analyzed their correlations with hub genes, providing a new perspective on the role of immunity, autophagy, and metabolism in ARDS.

## Data availability statement

The original contributions presented in the study are included in the article/**Supplementary Material**. Further inquiries can be directed to the corresponding author.

## Ethics statement

Ethical approval was not required for the study involving humans in accordance with the local legislation and institutional requirements. Written informed consent to participate in this study was not required from the participants or the participants’ legal guardians/next of kin in accordance with the national legislation and the institutional requirements.

## Author contributions

FX and FG contributed to the conception of the protocol. FX contributed to data acquisition. FX wrote the first version of the manuscript. All authors contributed to the article and approved the submitted version.
