# Supplementary material for: Development of genomic phenotype and immunophenotype of acute respiratory distress syndrome using autophagy and metabolism-related genes
Source: Front Immunol. 2023 Oct 23;14:1209959. doi: 10.3389/fimmu.2023.1209959 (PMC10626539; doi:10.3389/fimmu.2023.1209959)
Supplement: Supplementary file 4 [file Table_4.docx]

**Table S4. GO enrichment results of differentially expressed genes of high and low risk groups in the integrated GEO data set**

| ONTOLOGY | ID | Description | p.adjust |
| --- | --- | --- | --- |
| BP | GO:0042119 | neutrophil activation | 1.00E-51 |
| BP | GO:0002283 | neutrophil activation involved in immune response | 1.04E-51 |
| BP | GO:0043312 | neutrophil degranulation | 6.21E-51 |
| BP | GO:0002446 | neutrophil mediated immunity | 4.34E-50 |
| BP | GO:0072593 | reactive oxygen species metabolic process | 8.10E-14 |
| BP | GO:0042742 | defense response to bacterium | 2.93E-12 |
| BP | GO:0002886 | regulation of myeloid leukocyte mediated immunity | 6.01E-11 |
| BP | GO:0006614 | SRP-dependent cotranslational protein targeting to membrane | 7.58E-11 |
| BP | GO:0019730 | antimicrobial humoral response | 1.40E-10 |
| BP | GO:0006613 | cotranslational protein targeting to membrane | 1.40E-10 |
| CC | GO:0034774 | secretory granule lumen | 6.49E-28 |
| CC | GO:0060205 | cytoplasmic vesicle lumen | 6.49E-28 |
| CC | GO:0031983 | vesicle lumen | 6.49E-28 |
| CC | GO:0042581 | specific granule | 1.43E-24 |
| CC | GO:0070820 | tertiary granule | 1.41E-19 |
| CC | GO:0035580 | specific granule lumen | 4.44E-16 |
| CC | GO:0005766 | primary lysosome | 5.66E-16 |
| CC | GO:0042582 | azurophil granule | 5.66E-16 |
| CC | GO:0030667 | secretory granule membrane | 4.33E-14 |
| CC | GO:0044391 | ribosomal subunit | 4.05E-13 |
| MF | GO:0003735 | structural constituent of ribosome | 3.80E-09 |
| MF | GO:0031720 | haptoglobin binding | 1.49E-08 |
| MF | GO:0043177 | organic acid binding | 1.71E-07 |
| MF | GO:0005344 | oxygen carrier activity | 1.71E-07 |
| MF | GO:0004601 | peroxidase activity | 1.71E-07 |
| MF | GO:0016209 | antioxidant activity | 2.79E-07 |
| MF | GO:0016684 | oxidoreductase activity, acting on peroxide as acceptor | 2.79E-07 |
| MF | GO:0019864 | IgG binding | 4.66E-05 |
| MF | GO:0035325 | Toll-like receptor binding | 7.00E-05 |
| MF | GO:0140104 | molecular carrier activity | 0.0001323 |

GEO: Gene Expression Omnibus；GO: Gene Ontology；BP: Biological Process；MF: Molecular Function；CC: Cellular Component
