# Supplementary material for: Development of genomic phenotype and immunophenotype of acute respiratory distress syndrome using autophagy and metabolism-related genes
Source: Front Immunol. 2023 Oct 23;14:1209959. doi: 10.3389/fimmu.2023.1209959 (PMC10626539; doi:10.3389/fimmu.2023.1209959)
Supplement: Supplementary file 5 [file Table_5.docx]

**Table S5. KEGG enrichment results of differentially expressed genes of high and low risk groups in the integrated GEO data set**

| ID | Description | p.adjust |
| --- | --- | --- |
| hsa05171 | Coronavirus disease - COVID-19 | 3.86E-07 |
| hsa03010 | Ribosome | 3.91E-07 |
| hsa04613 | Neutrophil extracellular trap formation | 1.27E-05 |
| hsa05140 | Leishmaniasis | 0.000908 |
| hsa05150 | Staphylococcus aureus infection | 0.000908 |
| hsa04666 | Fc gamma R-mediated phagocytosis | 0.000908 |
| hsa05221 | Acute myeloid leukemia | 0.001148 |
| hsa04380 | Osteoclast differentiation | 0.001807 |
| hsa05134 | Legionellosis | 0.001807 |
| hsa04145 | Phagosome | 0.007715 |

GEO: Gene Expression Omnibus；KEGG: Kyoto Encyclopedia of Genes and Genomes
