# Supplementary material for: Development of genomic phenotype and immunophenotype of acute respiratory distress syndrome using autophagy and metabolism-related genes
Source: Front Immunol. 2023 Oct 23;14:1209959. doi: 10.3389/fimmu.2023.1209959 (PMC10626539; doi:10.3389/fimmu.2023.1209959)
Supplement: Supplementary file 6 [file Table_6.docx]

**Table S6. GSEA enrichment results of differentially expressed genes of high and low risk groups in the integrated GEO data set**

| ID | NES | p.adjust |
| --- | --- | --- |
| HALLMARK_ALLOGRAFT_REJECTION | 2.199573 | 5.56E-10 |
| HALLMARK_E2F_TARGETS | -2.3284 | 5.56E-10 |
| HALLMARK_G2M_CHECKPOINT | -2.16581 | 5.56E-10 |
| HALLMARK_HEME_METABOLISM | 2.360477 | 5.56E-10 |
| HALLMARK_INFLAMMATORY_RESPONSE | 2.328006 | 5.56E-10 |
| HALLMARK_INTERFERON_ALPHA_RESPONSE | 2.372642 | 5.56E-10 |
| HALLMARK_INTERFERON_GAMMA_RESPONSE | 2.312702 | 5.56E-10 |
| HALLMARK_MYC_TARGETS_V1 | -2.61593 | 5.56E-10 |
| HALLMARK_OXIDATIVE_PHOSPHORYLATION | -2.30463 | 5.56E-10 |
| HALLMARK_KRAS_SIGNALING_UP | 2.084548 | 6.09E-10 |

GEO: Gene Expression Omnibus；GSEA: Gene set enrichment analysis
