# Supplementary material for: Development of genomic phenotype and immunophenotype of acute respiratory distress syndrome using autophagy and metabolism-related genes
Source: Front Immunol. 2023 Oct 23;14:1209959. doi: 10.3389/fimmu.2023.1209959 (PMC10626539; doi:10.3389/fimmu.2023.1209959)
Supplement: Supplementary file 9 [file Table_9.docx]

**Table S9. Basic information of patients with acute respiratory distress syndrome by immunophenotype**

|  | Cluster1 | Cluster2 |
| --- | --- | --- |
| ARDS | 22 | 35 |
| Normal | 0 | 0 |

ARDS: Acute Respiratory Distress Syndrome
