# Supplementary material for: Development of genomic phenotype and immunophenotype of acute respiratory distress syndrome using autophagy and metabolism-related genes
Source: Front Immunol. 2023 Oct 23;14:1209959. doi: 10.3389/fimmu.2023.1209959 (PMC10626539; doi:10.3389/fimmu.2023.1209959)
Supplement: Supplementary file 10 [file Table_10.docx]

**Table S10. Basic information of patients with acute respiratory distress syndrome by disease subtype**

|  | Group1 | Group2 |
| --- | --- | --- |
| ARDS | 38 | 19 |
| Normal | 0 | 0 |

ARDS: Acute Respiratory Distress Syndrome
